# Pulsed Electron Double Resonance in Structural Studies of Spin-Labeled Nucleic Acids

**Published:** 2013

**Authors:** O. S. Fedorova, Yu. D. Tsvetkov

**Affiliations:** Institute of Chemical Biology and Fundamental Medicine, Siberian Branch, Russian Academy of Sciences, Lavrentyev Ave., 8, Novosibirsk, 630090; Institute of Chemical Kinetics and Combustion, Siberian Branch, Russian Academy of Sciences, Institutskaya Str. 3, Novosibirsk, 630090

**Keywords:** pulsed electron double resonance (PELDOR), spin-labels, DNA, RNA, oligonucleotides

## Abstract

This review deals with the application of the pulsed electron double resonance (PELDOR) method to studies of spin-labeled DNA and RNA with complicated spatial structures, such as tetramers, aptamers, riboswitches, and three- and four-way junctions. The use of this method for studying DNA damage sites is also described.

## INTRODUCTION. FOUNDATIONS OF THE PELDOR THEORY

Pulsed electron double resonance (PELDOR), or DEER (abbreviation used for pulsed electron double resonance or double electron-electron resonance; the former abbreviation is used hereinafter), which was developed at the Institute of Chemical Kinetics and Combustion (Siberian Branch of the Russian Academy of Sciences) in 1981 [1], is today considered the most popular EPR method. It is widely used in structural studies of systems containing paramagnetic centers.

Reviews that cover the PELDOR theory and provide examples of its application to structural investigations are published virtually every year. Only the works that have been published over the past 5 years are mentioned here [2–8]. The most significant success in the research into biomacromolecules using PELDOR has been achieved undoubtedly due to the development of effective methods of site-directed spin labeling. Many of these works cover investigations of DNA and RN A in specific biochemical systems. However, these efforts are only briefly discussed in the reviews [2–8] and only in combination with the other examples of PELDOR application. This review aims to present the results of PELDOR application in structural studies of the important classes of biomacromolecules, DNA and RN A, in a systematic manner. The review covers studies that were carried out mostly during the period between 2003 and the first half of 2012.

Two spin labels are typically introduced into molecules for PELDOR investigations. Nitroxide radicals are usually used as labels. The dipole and exchange magnetic interactions between the labels contain information regarding the distances between the labels, their mutual orientation, the aggregation and complexation of labeled molecules, and the spatial distribution of the spin labels in the investigated system. What makes PELDOR so important and unique is the possibility of using it in *systems *of *randomly oriented *particles.

Let us provide the most important information on the PELDOR theory required for the analysis of PELDOR results in this review. Detailed information regarding the PELDOR theory can be found in [2, 4, 9], and a description of methodological questions and PELDOR spectrometers can be found in [10].

The magnetic dipole–dipole interaction between the A and B spin labels is determined by a dipole frequency [4, 9, 11]:

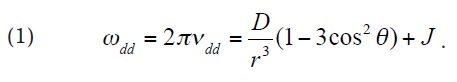



**Fig. 1:**
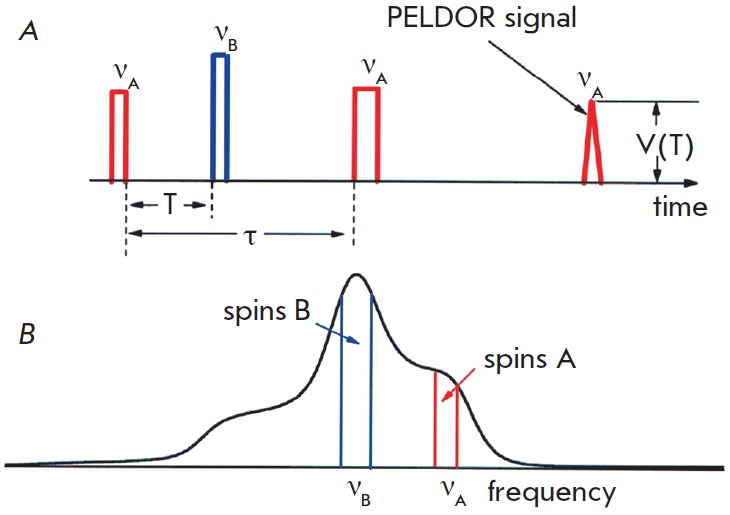
(A) 3p PELDOR pulse sequence. The spin echo signal is the result of the action of two pulses at frequency *v*_A_. When the third pumping pulse at *ν*_B_ acts on the spin system, the PELDOR effect arises and can be registered as a time scale function, V(T). (B) The positions of the pumping (B) and registration (A) pulses in the EPR frequency scale

Here, *D *= 327 rad nm^3^/μs is the dipole–dipole interaction constant, *r *is the interspin distance, θ is the angle between the direction of the external magnetic field and the vector connecting the spins, and *J *is the exchange integral. Three-pulse PELDOR (3pPELDOR) is used to determine the dipole frequency and, hence, the distance between the spins. This sequence is shown in *Fig. 1A* and consists of 2 types of pulses at the frequencies *ν*_A_ and *ν*_В_. Pulses π/2 and π at the frequency *ν*A acting upon the spins A in the EPR spectrum (*Fig. 1B*) are used to form the spin echo signal, which is then used to detect the PELDOR effect. The interval τ between the pulses at the frequency *ν*_A_ is fixed. The pumping pulse π at the frequency *ν*_В_ , which acts on the spins B with a delay *T *counted from the first π/2 pulse, lies in this interval. The pumping pulse changes the orientation of the spins B, resulting in a change in the dipole interaction between the spins A and B. This change is recorded as the decay of the amplitude of the spin echo signal,* V (T)*, when the delay *T *changes in the interval 0 – τ. The time trace V(T) is modulated at the frequency *ν_dd_*, which allows one to determine the interspin distance* r*. Modulation in the PELDOR time trace was first observed and investigated in [12, 13].

A four-pulse PELDOR sequence (4рPELDOR) is also used. Here, an echo signal is formed under the influence of three pulses π/2, π, π at frequency *ν*_A_ and change in it occurs due to the π pumping pulse, which is applied in the interval between the second and third pulses at *ν*_B_.

The PELDOR time trace for a randomly oriented pair of spin labels with a fixed *r *under the approximation of short microwave pulses is described by the following relationship [4, 9]:

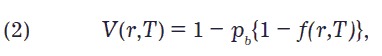



where

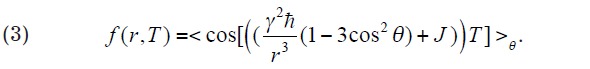



**Fig. 2:**
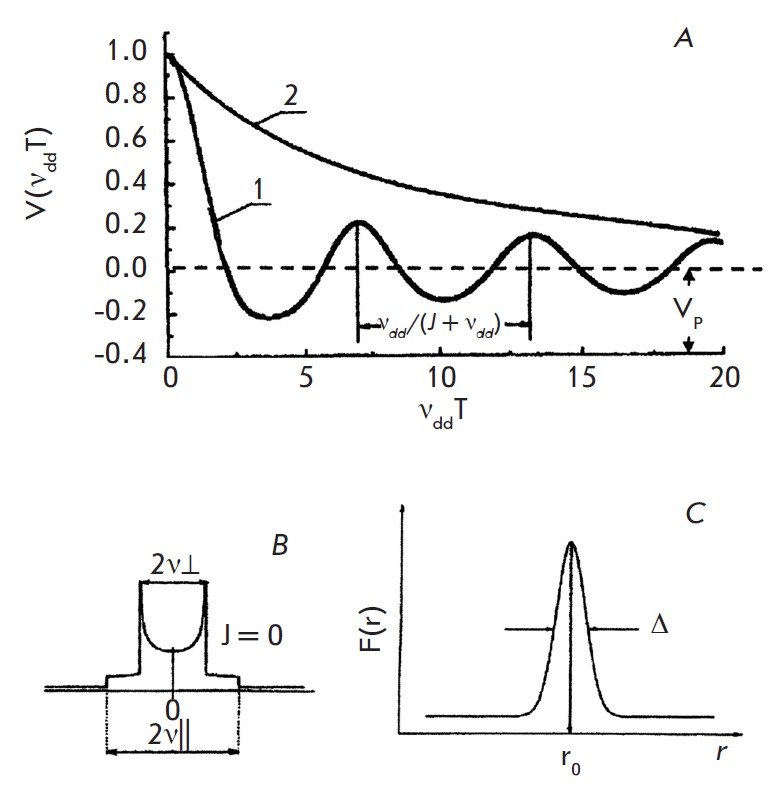
*А *– The PELDOR time trace *V(T) *modulated at the *(ν_dd_+J) *frequency (curve *1*) and its limiting value *V_p_*at* T→*∞*. *The exponential PELDOR time trace for paramagnetic particles uniformly distributed in the volume (curve *2*).* B *– Fourier conversion of the modulated PELDOR time scale (*J = 0*). *C *– The distance distribution function (distance spectrum) *F(r), r_0_*– distance in radical pairs, Δ – half width at half height of *F(r) *function

Here, *p_b_*is the probability of rotation of one of the spins in a pair when the pumping pulse is applied: <...>_θ_ denotes the averaging on the θ angle. The integration of (2) and (3) yields a decreasing function modulated by attenuating oscillations at frequency *ν_dd_*(*Fig. 2A*, curve* 1*). A Fourier analysis of this PELDOR time trace yields a so-called Pake dublet (*Fig. 2B*), which allows one to obtain data on the distance *r *and the exchange integral* J* [4, 9], since

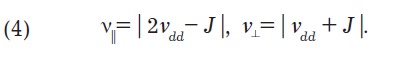



At rather large time periods *(Т→*∞*), *the function* V*(*r*,*T*) approaches the limit value *V_Р_*(*Fig. 2A*),

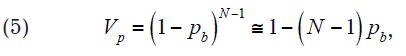



whose value is determined by the number (*N*) of dipole–dipole interacting spins [4, 9], which enables to determine the number of spin-labeled molecules in the aggregates and the complexes.

The maximum distance that can be measured using PELDOR is determined as the maximum time of the phase relaxation in the spin system under study and typically lies in a range of ~ 8nm. The minimum distance depends on the duration of the pumping pulse and is typically ~ 1.5 nm under optimal experimental conditions [4].

The distance between a spin-label pair can remain unfixed for different reasons. In this case, a distance distribution function *F(r) *between the labels (distance spectrum) is introduced, which is determined as* F*(*r*) = *dn(r)/dr, *where *dn(r) *is the fraction of spin-label pairs with the distance between the labels in a pair in the range between *r *and *r*+*dr. *In the case of continuous distance distribution, the function describing the PELDOR time trace can assume the following form [14]:

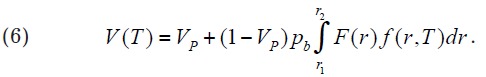



The limits of integration *r*_1_ and *r*_2_ in (6) restrict the physically reasonable range of distances between spin labels. Expression (6) is a first-kind Fredholm equation whose solution is unstable due to the inaccuracies in the experimental value *V*(*T*). The calculation of *F*(*r*) basically reduces to a solution of the inverse problem using the Tikhonov regularization techniques [15]. Meanwhile, one should bear in mind that the unstable properties of the solution to the equation are not eliminated. The methods for estimating the distance distribution function in radical pairs from experimental PELDOR data were developed in [16–19], and the method for three spin labels was shown in [20]. The program for estimating* F(r) *using the PELDOR time trace was provided in [21]. The maximum of the function *F(r)* corresponds to the distance between the spin labels *r_0_*, and its width Δ corresponds to the spatial distribution of the distances (*Fig. 2B*). Let us note that in accordance with (5) and (6), estimation of *F(r) *enables to independently determine* N *(the number of spins in a group).

Two types of dipole interactions exist in real systems containing spin groups: those between the paramagnetic centers within a group (*V(T)_INTRА_*) and those between the paramagnetic centers of different groups (*V(T)_INTЕR_*). The dipole interaction within the pairs or inside specific groups of spin labels was discussed above. If these interactions are considered independent, then the entire function describing the time trace *V(T) *can be written as [4, 9]:

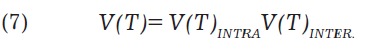



In most cases, PELDOR is used to investigate systems of spin labels or groups of labels which are uniformly distributed over a volume. The PELDOR time trace for paramagnetic centers randomly distributed over a three-dimensional space can be described using the exponential function [4]

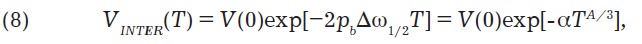



where Δω_1/2_ = 8.2 ・ 10^-13^·*C, *cm^3^·s^-1^ is the dipole bandwidth, and *C *is the concentration of the paramagnetic centers (in cm^-3^). In general, the α and *А *values depend on the spatial dimensions. For instance, A = 3 for a 3-dimensional space (*Fig. 2A*, curve *2*), A = 2 for a plane, and A = 1 for a line [4, 9]. The α and *А *values can be also calculated for more complex situations of spatial distribution of paramagnetic centers [22]. The comparison of the experimentally determined and the estimated α and *А *values opens the doors to investigating the features of spatial distribution using PELDOR.

The techniques based on recording the exponential time trace *V(T)_INTER_*and its dependence on the concentration of paramagnetic centers, which enable separation of *V(T)_INTER_*and *V(T)_INTRA _*for further analysis, have been devised [24, 24].

**Fig. 3:**
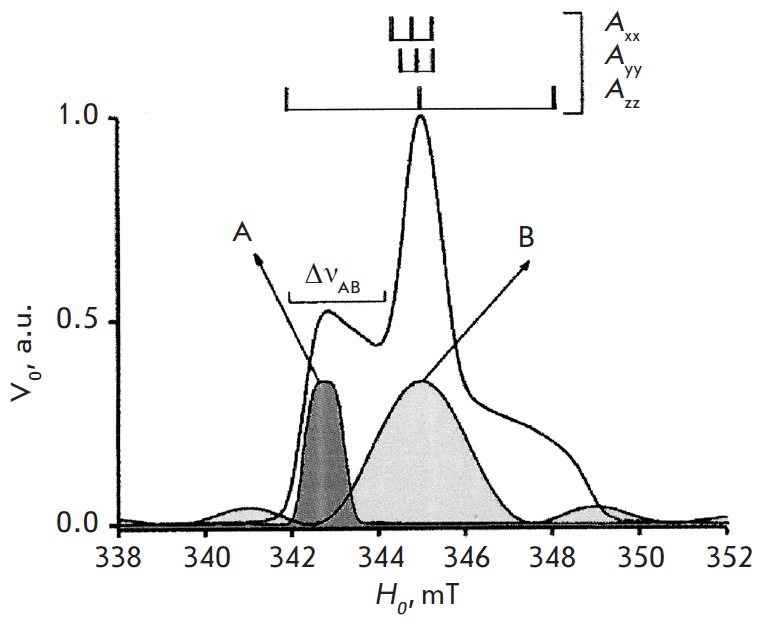
Schematic representation of the experiments on orientation selectivity measurements for the nitroxide spin labels. The EPR spectrum shape is the function of the main anisotropic hyperfine tensor elements A_xx_, A_yy_, A_zz_. A and B denote the recording and pumping pulses. PELDOR time traces *V(T) *recorded at different Δ*ν_AB_* fixed on the A_zz_ component of the EPR spectrum

The pulses A and B act selectively in the different narrow frequency ranges of the EPR spectrum. The orientational selectivity of the effect of microwave pulses on the spin system emerges if the value of the anisotropy of the magnetic-resonance parameters of the paramagnetic centers is relatively high (like that for nitroxide spin labels). This selectivity means that the radicals differently oriented in space are excited to different extents by the echo forming pulses and by the pumping pulse. The theoretical analysis and the experimental data demonstrated that the data on the mutual orientation of spin labels and their orientation relative to the vector *r, *which connects a label pair, can be obtained from PELDOR time traces. The measurements should be carried out while varying the positions of the A and B pulses in the spectrum or Δν_АВ_ [25–27]. The scheme for conducting these experiments for a typical EPR spectrum obtained for a nitroxide spin label in the X-band is shown in *Fig. 3*.

The contemporary PELDOR theory and experimental techniques allow one to obtain and study the structure and properties of numerous biologically important molecules. The results of DNA and RN A studies using this method are discussed below.

## PELDOR STUDY of SPIN-LABELED DNA AND RNA

**Spin labels for DNA and RNA**

The development of site-directed spin labeling has enabled to elaborate a wide range of EPR spectroscopy applications in biochemistry and biophysics. These comprise the determination of the elements of the secondary and tertiary structures of membrane proteins, including the environmental influence; research into the orientation and motion of separate protein fragments under physiological conditions; detection of the conformational transitions in the functioning of membrane protein complexes; etc.

These investigations are usually performed using conventional stationary EPR methods and are published regularly in a series of collected articles entitled *Biological Magnetic Resonance *edited by L. Berliner *et al. *(a total of 28 books had been published by 2011). Let us discuss only the articles dealing with the application of PELDOR for the structural investigation of DNA and RN A. The results of the first experiments in this field were published in volumes 19 and 21 of this series [28, 29].

The elaboration of efficient methods for the synthesis of site-directed spin-labeled biologically important compounds was the most significant stage in these experiments and made them feasible. The reviews [30–32] discuss a series of methods that resolve this problem for nucleic acids and oligonucleotides. A brief review devoted to spin labeling of DNA and RN A has recently been published [33].

2,2,6,6-Tetramethylpiperidine-N-oxyl (TE MPO), 2,2,5,5-tetramethyl-pyrrolin-1-oxyl-3-acetylene (TPA), and 2,2,6,6-tetramethyl-3,4-dehydropiperidin- N-oxyl-4-acetylene (TE MPA) are the most popular spin labels used to label proteins and nucleic acids:

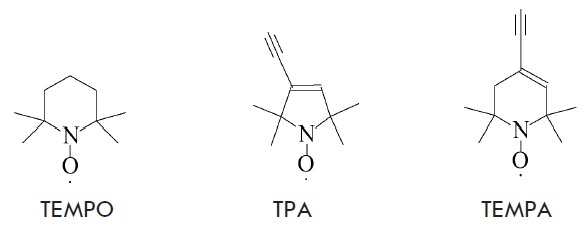



An unpaired electron in these molecules is localized on the N-O-fragment.

In the first studies, the label was introduced into the C5 position of the uracil residue of the nucleic acid [34]. Since the limitation of the conformational mobility of spin labels increases accuracy in distance determination using PELDOR, rigid linkers have recently been used to introduce spin labels into nucleic acids. The Sonogashira method, which is based on the replacement of iodine in the organic compound with an alkynyl residue, is one of such methods commonly used today [35, 36]. The reaction is catalyzed by Pd(II) and Cu(I) salts. For the nucleic acids, TPA and TE MPA residues are introduced into the C5 position of iodouridine via reaction A [34, 37–40] giving rise to adducts.

Reaction A:

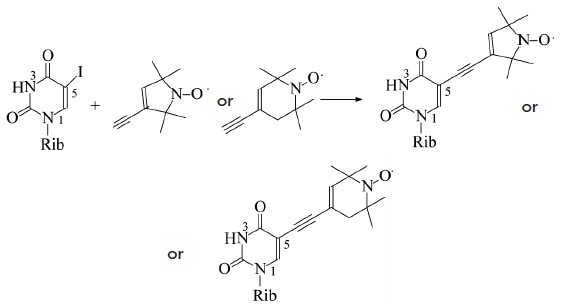



where Rib is the ribosomal residue.

This technique enables to introduce spin labels both into the monomers used in the phosphoramidite method for oligonucleotide synthesis and into the complete ribo- and deoxyribo-oligonucleotides. In the course of the ribooligonucleotide synthesis, the TPA spin label is also introduced into the adenine and cytosine residues containing protected amino groups via the Sonogashira reaction [41].

Azide-alkyne cycloaddition, known as “click chemistry,” is also commonly used today to introduce spin labels into oligonucleotides [42]. In this case, the spin label is introduced into the oligonucleotide via the Cu(I)-catalyzed reaction between the acetylene group (incorporated into the heterocyclic base at the C7 position of 7-deazaadenine or the C5 position of uracil) and 4-azido-TE MPO in solution [43], or in the course of solid-phase synthesis of oligonucleotides [44] coupled with adduct formation.

Reaction B:

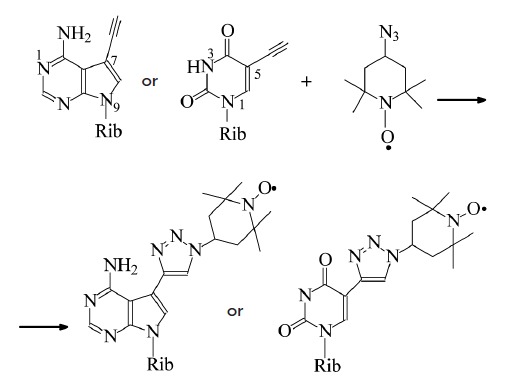



This reaction is stereospecific, characterized by a high yield, and is used to synthesize spin-labeled DNA and RN A.

**Linear duplexes of nucleic acids**

The structures of 12-bp (base pairs) duplexes (RN A1) and 15-bp - duplexes (RN A2) were investigated in work [45]. The TE MPO labels were introduced into the 2’-NH_2_-groups of ribose in uridine (**U**) residues via the reaction with TE MPO isothiocyanates:

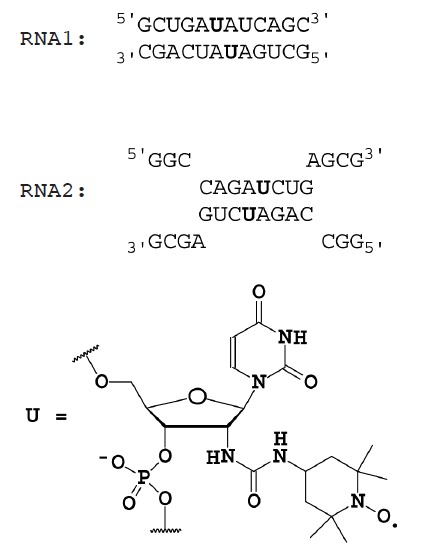



The modulated PELDOR time trace was recorded only for RN A1. The distance between the spin labels (3.5 ± 0.2 nm) was determined by Fourier analysis. Only the exponential PELDOR time trace was recorded for RN A2, which was testament to the uniform distribution of the spin labels. This means that no duplexes were formed between the spin-labeled oligonucleotides of RN A2 in a water solution (buffer 0.1 M NaCl, 0.01 M Na-phosphate, 0.1 mM Na2EDTA, pH 7.2) at a concentration of 0.3 mM. This fact suggests that intramolecular hairpin structures could have formed, the process being predominant over the bimolecular process of duplex formation.

**Fig. 4:**
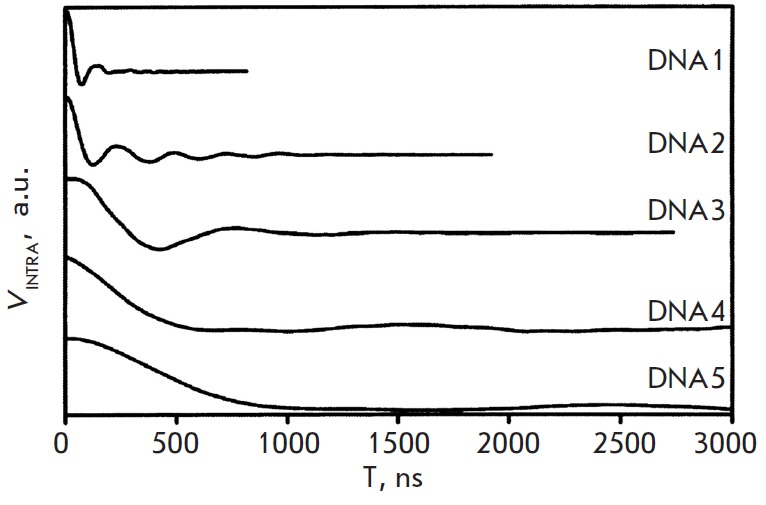
PELDOR time traces *V_INTRA_*for five spin-labeled DNA [46]. (Reproduced by permission from the American Chemical Society:[Schiemann, O., Piton, N., Mu Y., Stock, G., Engels, J.W., Prisner, T.F. (2004) *Am. Chem. Soc*. 126, 5722-5729], copyright 2004)

The distances between the TPA spin labels introduced via the reaction A into the residues of 2’-deoxyuridine (U) of the DNA duplex helices were determined using the 4рPELDOR [46]. The labels were introduced into the U residues located at 5 different positions in the duplex, so that the number of bases between the labels,* n*, was different: *n *= 0, 2, 8, 10, 12, respectively, for the DNA1-DNA5; for instance (spin-labeled **U **residues are in shown in bold):

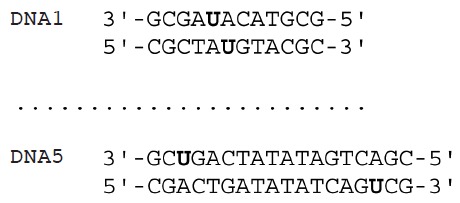



**Fig. 5:**
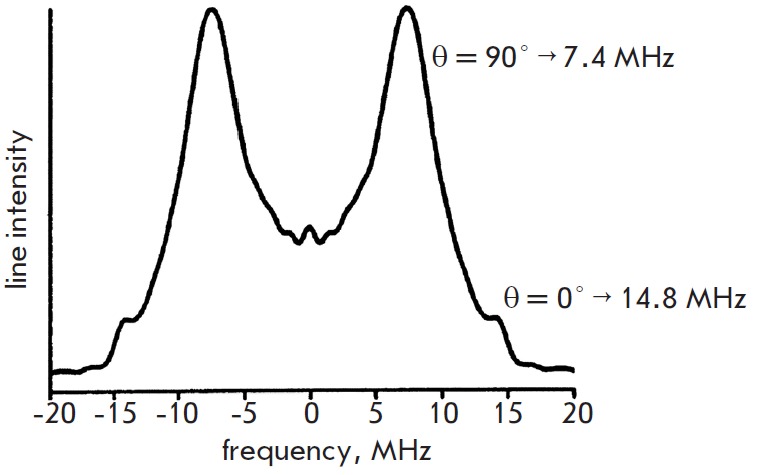
Fourier spectrum of *V_INTRA_* for DNA-1. Lines at *ν_┴_ (θ = 90˚) *and *ν_//_ (θ = 0˚). *[46]. (Reproduced by permission from the American Chemical Society:[Schiemann, O., Piton, N., Mu Y., Stock, G., Engels, J.W., Prisner, T.F. (2004) *Am. Chem. Soc*. 126, 5722-5729], copyright 2004)

Frozen (35 K) aqueous buffer solutions of the duplexes with the addition of 20% ethylene glycol for vitrification were investigated. The modulation of the PELDOR time trace was recorded for all DNA molecules (*Fig. 4*). The period of beating of the time traces increased with an increase in the distance between the spin labels. The Fourier spectra in all the cases had the shape of Pake doublets. An example of this doublet for DNA1 is shown in *Fig. 5*. The lines in this doublet (at a frequency of 7.4 and 14.8 MHz) correspond to a parallel (θ = 0^0^) and a perpendicular (θ = 90^0^) orientations of the vector connecting the spin labels *r *relative to the direction of the external magnetic field. The distance between the labels can be determined using the ratios (1) and (4). In this case, *r *= 1.92 nm and the *J*-exchange integral is equal to 0.

**Fig. 6:**
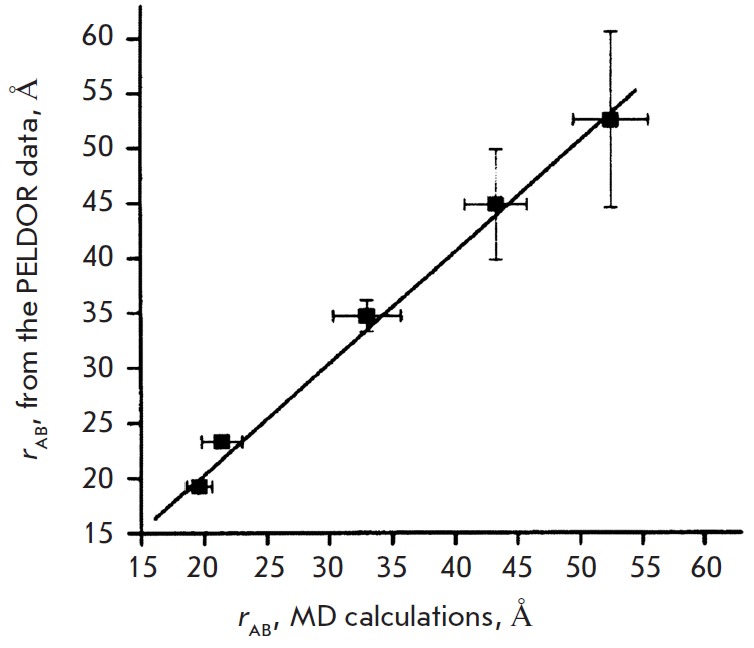
Correlation of the distances *r_AB_* obtained by PELDOR experiment and MD calculations [46]. (Reproduced by permission from the American Chemical Society:[Schiemann, O., Piton, N., MuY., Stock, G., Engels, J.W., Prisner, T.F. (2004) *Am. Chem. Soc*. 126, 5722-5729], copyright 2004)

The distances determined from the Fourier spectrum for DNA2-DNA5 were equal to 2.33, 3.47, 4.48, and 5.25 nm, respectively. These distances for the investigated spin-labeled DNA were calculated using molecular dynamic (MD) simulations [46]. The results of a comparison of the theoretical and experimentally obtained values listing all probable errors are shown in *Fig. 6* The correlation coefficient of these results is equal to 0.997, which is believed [46] to support the existence of B-conformation in the duplex helix in frozen aqueous solutions. A detailed comparison of the PELDOR and FRET (fluorescence resonance energy transfer) [46] methods demonstrated that these methods complement each other in the investigations of spin-labeled systems.

Six TPA-labeled RN A duplexes were synthesized [41]. A sufficiently deep modulation recorded for the PELDOR time traces enabled to calculate the distribution function, *F(r), *and accurately determine the distances in the duplexes, which lie in a range from 1.93 ± 0.12 to 3.87 ± 0.13 nm, depending on the number of base pairs between the labels. The comparison of the experimental results in [41] and [46] with the results of a measurement of the distances in DNA demonstrated that given an identical number of base pairs, the distances between the labels in DNA and RN A located in different helices of the duplex are different. Hence, when the labels are located in bases at a distance of 10 bp, these values are equal to 4.48 ± 0.5 nm and 3.87 ± 0.13 nm in DNA and RN A, respectively [41]. This difference cannot be accounted for by a measurement error; it corresponds to two different conformations: the A-form in RN A and the more stretched B-form in DNA. It turned out that the results obtained were in close agreement with those obtained by MD simulations; the correlation coefficient was 0.976 [41]. The authors believe that this result shows that the DNA and RN A duplexes maintain their conformations in frozen (40 K) aqueous phosphate buffer solutions.

In the paper of Q. C ai et *al.* [47], the TPA labels were ;introduced via a methylene linker not into a heterocyclic base but into the phosphorothioate groups at specific positions of the sugar-phosphate backbone. The duplex formed from polynucleotides labeled at different positions enabled one to measure the distances between arbitrary points of DNA duplexes. The samples prepared in this manner (the measurements were taken at 50 K in frozen aqueous solutions of DNA duplexes) were used to determine eight interspin distances in a 12 bp DNA duplex by PELDOR on the basis of the position of the maximum values of the distance spectrum. The minimum and maximum distances were equal to 2.56 and 3.88 nm, respectively, for DNA. According to the authors, this method of labeling is not limited by the polynucleotide length [47]. The data for a 68bplong DNA fragment containing labels located opposite each other at a distance of 9 nucleotides from one end of the duplex are provided in this article. The distance measured using PELDOR (2.52 nm) was equal to that obtained with MD simulation (2.5 nm).

**Fig. 7:**
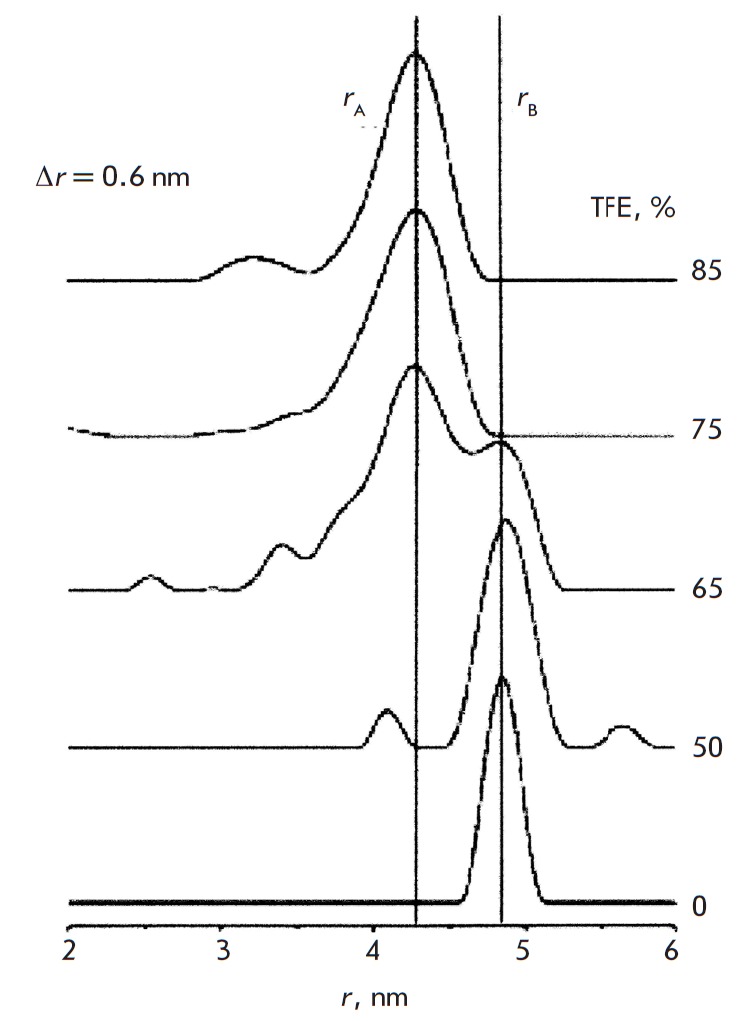
PELDOR-measured distance spectrum change after TFE was added to a water solution of a 4; 18’ labeled DNA duplex. [49]. (Reproduced by permission from John Wiley & Sons, Inc.: [Sicoli, G., Mathis, G., Delalande, O., Boulard, Y., Gasparutto, D., Gambarelli, S. (2008) *Angew. Chem. Int. Ed*. 47, 735-737], copyright 2008)

Q. C ai et *al. *[47] also compared the results of the PELDOR measurement with those calculated using NMR spectroscopy, which takes into account the probable conformations of the DNA under study, and found an excellent correlation (*R*^2^= 0.98) between the PELDOR and NMR measurements . They believe that the method proposed for label introduction can be widely used in structural studies of DNA– and RN A–protein complexes.

An investigation very similar to [47] is described in [48], where spin labels were introduced into phosphorothioate groups of RN A; six sets of interspin distances ranging from 2.5 to 4.72 nm were compared to the Xray data. A positive correlation between these measurements was found (*R*^2^ = 0.97). This fact indicates that the introduction of a label does not significantly alter the RN A structure.

It was shown in the investigations described above that the methods developed for spin labeling of linear DNA and RN A duplexes allow an appreciably accurate (~ 1%) determination of the distance between the spin-labeled nucleotides. The strict correlation between the PELDOR and MD results is of significance. The MD simulations were typically carried out at room temperatures and for aqueous solutions; the PELDOR measurements were carried out using rapidly frozen vitreous solutions. It can be concluded that the conformations existing in DNA and RN A molecules at room temperature is instantaneously fixed as the molecules froze. This fact substantiates future PELDOR studies of DNA and RN A in different environments or in the course of various interactions and reactions.

The inter-nucleotide distance changes in a transition from the B- to the A-conformation of DNA. This change was recorded and studied using PELDOR [49]. In this case, spin-labeled complementary DNA duplexes were investigated:

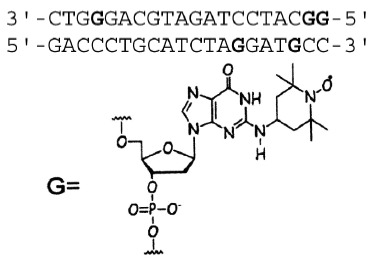



The 4-amino-TE MPO label was introduced into the N2 atom of the guanine residue located either in the same (top) helix (positions (4; 19), (4; 20)) or in both helices. In the latter case, the labels occupied the positions (4; 14΄) or (4; 18΄) in different helices.

The transition between the B and A forms of DNA occurs in polar media. The spin-labeled DNA duplexes were investigated at 60–70K in an aqueous buffer with the addition of 10 vol. % glycerol (as a cryoprotectant). Trifluoroethanol (TFE) was added to stimulate the В → А transition. The changes in the distance spectrum in response to the changes in the volumetric content of TFE are shown in *Fig. 7*. The B-form is converted into the A-form at a TFE concentration exceeding 70%. The difference between the average distances for the A- and B-forms is 0.8 nm. The interspin distances for all the investigated samples of DNA in the A- and B-forms are shown in *Table 1*. The MD simulation values for the distances between the oxygen atoms of the >NO groups of the spin labels in the investigated duplexes are also provided for comparative purposes.

**Table 1:** Experimental and calculated distances between the spin labels (nm) for the A- and B-form of DNA

DNA duplex	PELDOR	B-form, O–O distance, MD calculation	PELDOR	A-form, O–O distance, MD calculation
(4;20)	5.6 ± 0.2	5.6 ± 0.3	4.8 ± 0.2	4.5 ± 0.4
(4;19)	5.1 ± 0.2	5.1 ± 0.3	4.6 ± 0.3	4.4 ± 0.4
(4;18')	4.9 ± 0.2	4.8 ± 0.4	4.3 ± 0.3	4.2 ± 0.4
(4;14')	3.2 ± 0.2	3.6 ± 0.3	2.8 ± 0.3	3.3 ± 0.3

According to [49], PELDOR sensitivity in distance determination in the nanometric range is considerably higher than that for any other method, such as stationary EPR or circular dichroism (CD). This fact justifies the investigation of the transitions between different conformers of the A- and B-forms of RN A and DNA under various conditions of molecular environment and polarity.

It was shown using an MD simulation [50] that localization of the TPA spin label in the major DNA or RN A groove results in a change in the mutual orientation of the base pairs in the molecule. This effect is less significant for the labels located in the minor groove. Nonetheless, the conformational changes that occur during the incorporation of the labels into DNA or RN A should be taken into account when interpreting the orientational effects in PELDOR.

**Fig. 8:**
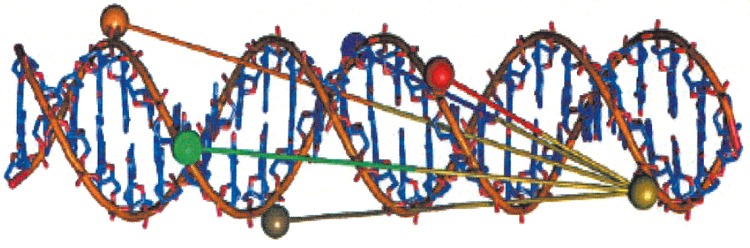
Molecular model showing the positions of spin label pairs for the distance measured in [52]. (Reproduced by permission from John Wiley & Sons, Inc.: [Ward, R., Keeble, D.J., El-Mkami, H., Norman, D.G. (2007) *Chem- BioChem. *8, 1957-1964], copyright 2007)

The distances between the spin labels in 4 hybrid DNA/RN A-duplexes were measured [51]. The TPA spin labels were incorporated into the heterocyclic bases in such a way that they were oriented towards either the major or the minor groove of the duplex. This allowed one to choose between the A- and B- conformations of the hybrid. The B-conformation was found in 50% of the cases, and the A-conformation typically occurred in the rest of the cases. During the interaction of sufficiently long DNA and RN A duplexes with proteins and membranes, formation of conformational bending and disruptions of the linear structure, as well as the emergence of differently spatially oriented short duplex segments, are likely to occur. This heterogeneous system was formed using mixtures of DNA duplexes of identical length with spin labels incorporated into different segments [52]. The spin labels in the present work were introduced into the 2’-amino groups of uridine residues in the DNA duplexes in a stepwise manner via a reaction with isocyanate TE MPO in such a manner that the interspin distances were equal to 9, 12, 15, 18 or 21 bp:

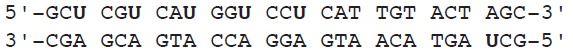



(spin-labeled nucleotides are shown in bold).

A total of five double spin-labeled duplexes were synthesized. The optimally prepared samples (DNA solutions frozen at 77 K were studied) for the X-band of the PELDOR spectrometer (relatively high signal-tonoise ratios, long relaxation time *Т_f_*≈ 8 μs) contained 12.5・ 10^-6^ mol l^-1^ of the DNA duplex in a 50% solution of deuterated ethylene glycol in D_2_О. The spin-label pairs (with the distances between them determined in different duplexes) are schematically shown in *Fig. 8*.

**Fig. 9:**
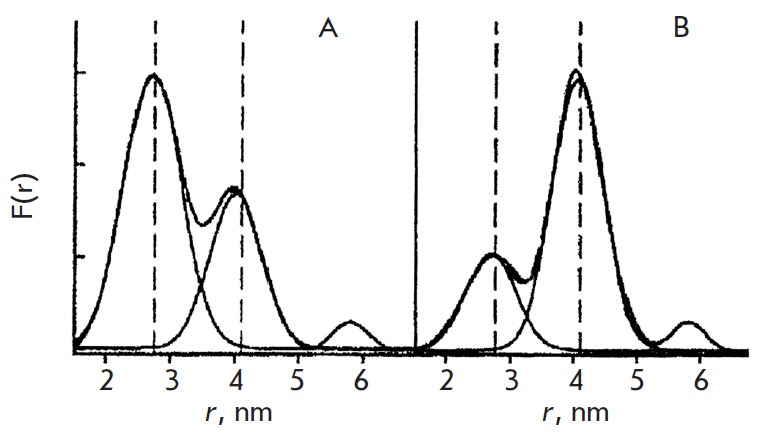
Investigation of spin-labeled DNA mixtures [52]. The distance distribution spectra are shown for a mixture of the DNA duplex labeled at distances 2.8 nm and 4.1 nm; A – at a 3:1 ratio; B – at a 1:3 ratio.(Reproduced by permission from John Wiley & Sons, Inc.: [Ward, R., Keeble, D.J., El-Mkami, H., Norman, D.G. (2007) *Chem-BioChem*. 8, 1957-1964], copyright 2007)

The PELDOR time trace was recorded; the distance spectrum estimated using Tikhonov’s regularization method were analyzed [15]. Six interspin distances in the range from 2.8 to 6.8 nm were determined. The results of the study of the mixtures containing the aforementioned spin-labeled duplexes are of particular interest (*Fig. 9*). It was ascertained [52] that for a mixture of two different duplexes, deconvolution of a composite function *F(r) *through the introduction of the distribution function in the form of a Gaussian curve for each duplex allows one to determine the average distance in each duplex and its concentration in the mixture with good accuracy. Meanwhile, certain difficulties arise during the analysis of the *F(r) *of mixtures containing large numbers of duplexes. These difficulties are apparently connected with both the inaccuracies and ambiguities in the solution of the inverse problem of recovery *F(r) *from the time trace *V(T)*, as well as with the probable transformations under the influence of such factors as stacking interactions in the complex mixture, which changes the spatial geometry of the duplexes [52].

The duplexes containing AA and TT mismatches (non-canonical pairs) were studied in [53]. Two TE MPO spin labels were introduced via the reaction of catalyzed cycloaddition (“click chemistry”) into one of the oligonucleotides on each side of the mismatches:

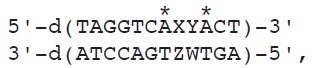



where А^*^ is 7-deazaadenozine containing the TE MPO spin label at C7; XY is the noncanonical pair dA x dA or dT x dT at positions 8 or 9 of the duplex. The distance between the spin labels in the canonical duplex, when XY/ZW = AT/TA, was 1.83 nm. The distance between the unpaired electrons in the duplexes **T**T/**T**A and **A**T/** A**A containing the **TT **or **AA **noncanonical pair at position 8 was 1.73 nm. The distances between the spin labels were 1.87 and 2.08 nm if the duplexes contained noncanonical pairs (A**T**/T**T **and A**A**/T**A**, respectively**)** at position 9. Thus, the introduction of a noncanonical pair into position 8 reduces the interspin distance, while the introduction into position 9 increases it as compared to the canonical duplex. Therefore, the DNA mismatch formation affects the structure of the adjacent base pairs, thus causing their convergence or divergence.

**Fig. 10:**
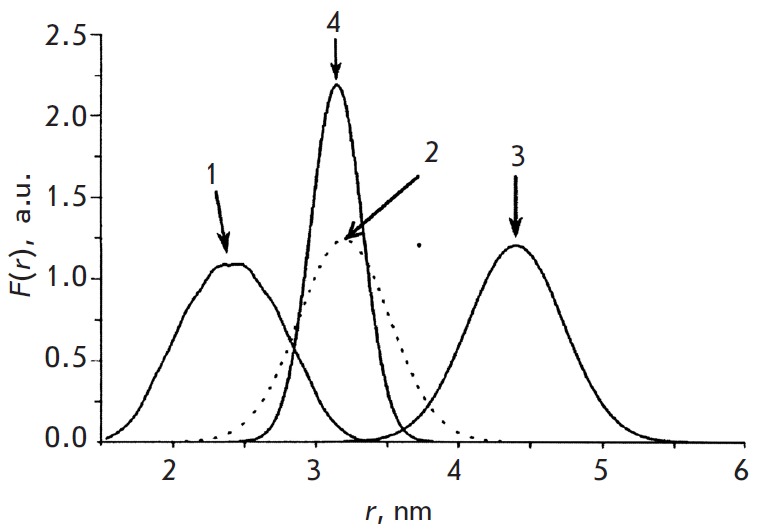
Distance distribution function *F(r) *for the trilabeled DNA duplex (arrows 1, 2, 3) and the same after treatment with Endo IV resulting to double-labeled DNA cleavage (arrow 4) [54]. (Reproduced by permission from John Wiley & Sons, Inc.: [Flaender, M., Sicoli, G., Aci-Seche, S., Reignier, T., Maurel, V., Saint-Pierre, C., Boulard, Y., Gambarelli, S., Gasparutto, D. (2011) *Chem-BioChem*. 12, 2560-2563], copyright 2011)

The DNA duplexes containing three TE MPO spin labels were also studied [54]. These Y labels were attached to the *C5 *atom of the uridine residue of the alkynyl-oligonucleotide using the “click chemistry” approach. Tetrahydrofuran X insertion (THF damage, see *Tables 3* and *5* below) was also introduced into one of the DNA strands in addition to two spin labels. Hence, the original system in the buffer solution contained three spin labels and one damaged site.

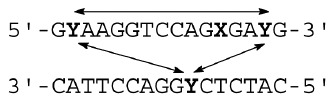



The PELDOR time traces in this 3-spin system were analyzed using the conventional procedure [21], which was modified for the 3-spin system [20]. As was expected, the distance spectrum in this system consisted of three lines with the peaks corresponding to the distances of 2.50, 3.15, and 4.60 nm and the widths of 0.05, 0.45, and 0.75 nm, respectively (*Fig. 10*).

The interaction between this DNA duplex and apurinic/ apyrimidinic endonuclease IV isolated from *Escherichia coli *(*EndoIV*) was also investigated [54]. DNA degradation is known to occur under the influence of* EndoIV *[55] at the apurinic/apyrimidinic (AP) sites and at the AP site analogs containing tetrahydrofuran (THF) residues instead of ribose. Duplex dissociation occurs at the THF residue, yielding a duplex containing only two spin labels and a DNA fragment with one label and a THF residue:

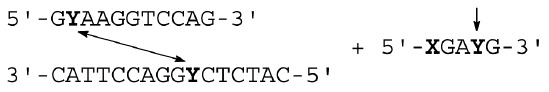



A single line (*Fig. 10*) was detected in the distance spectrum after denaturation during the investigation of the PELDOR time traces (with the maximum at 3.20 nm and a width of 0.75 nm), which was attributed to the spin-labeled duplex that remained after the degradation.

The results obtained demonstrate the potential for using PELDOR in the investigation of 3-spin DNA systems and, more importantly, expand the range of systems that can possibly be used for the analysis of the mechanisms of interaction between DNA and proteins and enzymes.

The data concerning the distances between the spin labels, as well as their mutual orientation, can be determined by studying the orientational selectivity using PELDOR spectroscopy. Orientation selectivity was investigated in [25–27, 56]. A special rigid spin label C was developed to study DNA [57]. It was rigidly bound to cytosine, which in turn was rigidly oriented and fixed by hydrogen bonds with the corresponding complementary base in the DNA structure:

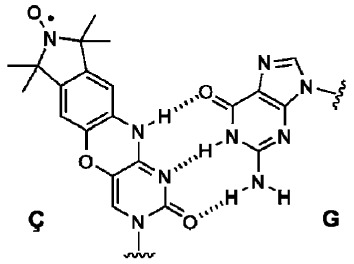



**Fig. 11:**
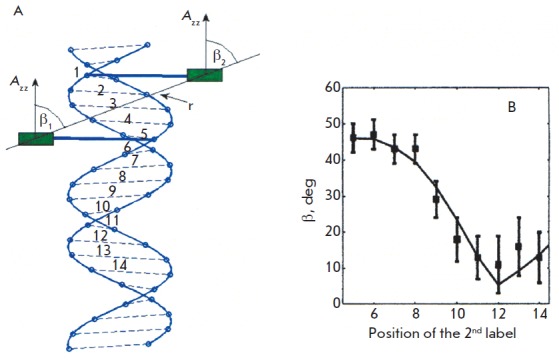
(A) Orientation of spin labels in the DNA structure. The spin labels are attached to the base pairs with the numbers 1 and 5. The principal axis of the hyperfine interaction tensor A_zz_ is normal to the labels planes; angles β_1_ and β_2_ are approximately equal. (B) Experimental and calculated dependence of β = β_1_= β_2_ on the position of the second label [27]. (Reproduced by permission from The American Physical Society: [Marko, A., Margraf, D., Cekan, P., Sigurdsson, S.T, Schiemann, O., Prisner, T.F. (2010) *Phys. Rev. E*. 81,021911], copyright 2010)

In accordance with the DNA structure, label planes are coplanar to the nucleotide pairs in DNA. Thus, the normal vectors to the label plane are parallel to each other (*Fig. 11*), and the β angle between the vector* r *connecting the labels and the normal to the plane of different labels will be the same. This finding made it possible to analytically determine this angle from the PELDOR time traces recorded at different frequencies of recording A and pumping B pulses; i.e., at different Δ*ν*_AB_. A detailed theory of this analysis and the experimental results are provided in [56]. The PELDOR time traces in the X-band of the EPR were measured for different frequency difference values (Δ*ν*_AB_) in a range from 90 to 40 MHz with a 10 MHz increment. The position of the second label in the investigated series of DNA samples varied from *N3 *to *N14* (*Fig. 11A*). The results obtained for angle β in DNA with varied positions of the labels are provided in *Fig. 11B*. It is clear that the angle estimated from the geometry of the DNA duplex fully corresponds to the experimentally obtained angle value. The obtained results create possibilities for investigating the orientation of spin labels in structures that are more complex than simple linear single- and double-stranded DNA and RN A.

The dynamic properties of nucleic acid molecules are of significance for understanding the kinetics and the mechanisms of cellular processes, such as replication and transcription, when DNA is twisted and bent upon the interaction with protein active centers. One of the urgent problems of modern biophysics is the investigation of the mechanisms of the molecular dynamics of nucleic acids. It was believed at the early stages of theoretical and experimental research that the dynamic properties of DNA duplexes can be described using the elastic cylinder model [58]. Various modern physical methods used to study the mechanisms of mobility of DNA helices [59–61] allow one to determine at least three types of possible motion, including change in the helical pitch without any changes in the helix radius (A), change in the helix radius with a constant helix pitch – elongation and twisting (B), and bending of the helix without changes in the radius and the pitch (C).

We have repeatedly mentioned before that the linewidth in the distance spectrum obtained in the PELDOR experiments at low temperatures in frozen glasses correlates with the spectrum of the possible conformational states of the spin system estimated for that same system using modern MD methods in liquids. This means that PELDOR provides snapshots of the dynamic situation for a given molecular system.

**Fig. 12:**
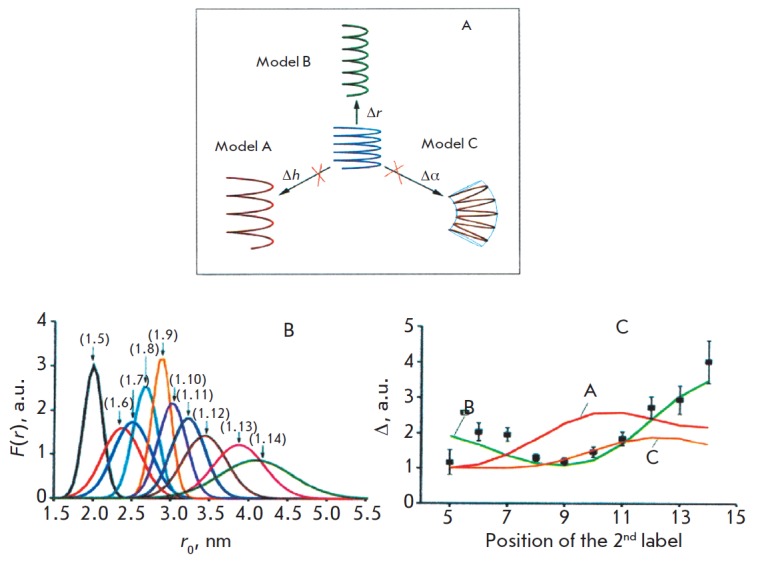
(A) Three models of cooperative motion in a double-stranded DNA molecule (see text). (B) Distance spectrum lines found from the X-band PELDOR data in a Gaussian approximation for* F(r). *The orientation selectivity for *C *labels in DNA duplexes was studied for spin labels pairs (shown in brackets). (C) Experimental spectra line widths, Δ = <ΔR>1/2, upon the distances between the labels and the theoretical calculations for different mobility types A, B, C. (text). The minimal Δ value corresponds to the distance between the 1 and 9 labels of DNA [62]. (Reproduced by permission from the American Chemical Society: [Marko, A., Denysenkov, V., Margraf, D., Cekan, P., Schiemann, O., Sigurdsson, S.Th., Prisner, T.F. (2011) *J. Am. Chem. Soc. *133, 13375- 13379], copyright 2011)

A. Marko *et al. *[62] used these features of PELDOR to separate the A, B and C mechanisms by studying the conformational flexibility of double spin-labeled DNA (20 nucleotides). Rigid spin labels C were incorporated into the duplex nucleotides. The paired labels were introduced into 10 duplexes in such a way that the position of one of them was fixed at one end of the duplex, and the distance R to the other labels was consistently increased for each pitch of the helix. The PELDOR measurements were carried out at the frequencies of the X-band (9 GHz) and the G-band (180 GHz). The PELDOR time traces and their dependency on Δ*ν*АВ for all the duplexes from (1.5) to (1.14) were measured; the linewidth Δ = <Δ*R*^2^>^1/2^ for *F(r) *was determined in the distance spectrum in the Gaussian approximation (*Fig. 12A*). The linewidth was determined by averaging the orientational selectivity data [26], which eliminate the correlation between the label orientations when the distances between them are measured. During the investigation of the orientational selectivity at different Δν_АВ_, the mutual orientations of the labels for all spin pairs were determined using the methods described in [25–27]. The theoretical computation of the modulated PELDOR time traces, the linewidth of the *F(r)* function, and the mutual orientation of the labels for different A, B, C motion models became an important phase of the investigation.

It was found that the relationship between the width of the spectrum lines and the position of a label can be used to interpret the experimental data (*Fig. 12B*) only for model B (helix winding). Twisting and stretching of the helix in this model can be determined from the data on spin-specific orientation obtained in the course of the experiments at 180 GHz. It was found that the angle variation between the relative orientations of the closest labels in the N–O bond was ±22˚.

As mentioned in [62], the results obtained completely agree with the model of cooperating fluctuations, the so-called model of “respiratory” movements of the DNA duplex, when the pitch of a helix remains constant, while the helix radius and the length of the DNA molecule vary in a correlated manner. According to the PELDOR data, the helix radius changes by 11% and the DNA length can change by ± 6%. All these PELDOR results correlate with the small angle X-ray scattering data (SAXS) [61] and with the results obtained via fluorescent microscopy [59] for short DNA polynucleotides. It should be mentioned that the wide variety of experimental approaches (the unique set of spin-labeled DNA, studies of the orientational selectivity, measurements carried out in various frequency ranges) used in this work [62] presumably for the first time demonstrated the potential of this method not only for structural studies, but for thorough studies of the dynamics of biomacromolecules as well.

Gd(III) [63–65] or Cu(II) [66] complexes have been recently suggested for use as labels. These labels are typically characterized by a rather complex EPR spectrum in polyoriented systems. However, when conducting measurements at high frequencies ~ 30 GHz (*K*a- range) and at cryogenic temperatures of~ 10 K, in the case of Gd(III) one line corresponding to the -. → +. transition prevails in the spectrum; this line is used in the PELDOR experiments. The structure of the duplex containing 14 base pairs was investigated using Gd(III) (Gd538 and Gd595) complexes as labels incorporated into the terminal thymidine molecules using the “click chemistry” method [65]:

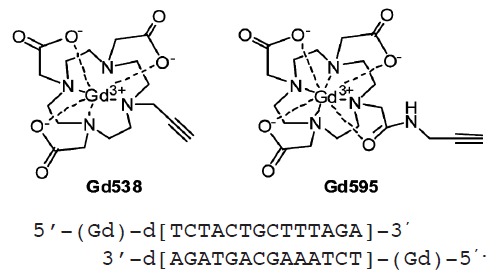



The measurements carried out by 4pPELDOR demonstrated that the distance between the ions in these DNA duplexes was approximately 5.9 ± 1.2 nm, while the width of the distance spectrum line was ~ 2 nm. The authors [65] believe that the use of these ions can increase the range of PELDOR-measured distances to ~ 10 nm, which is significant in the case of the conformations of complex biomolecules. A relatively large distance (1.2–1.5 nm) between the ions and the position at which they attach to the investigated molecule results in a wide distance spectrum and a decrease in the measurement accuracy due to the mobility of these labels. This is an obvious drawback of these labels in comparison to nitroxide labels. It should be mentioned that a number of features of the PELDOR analysis methods for Gd(III) and C u(II) were thoroughly examined in [65] and [66].

**Nonlinear duplexes and tertiary structures of DNA and RNA**

The secondary structure of DNA and RN A not only can appear as a linear helix, but can also have more complex configurations related to the tertiary structure of biopolymers. Relatively long 180^о^ bent singlestranded RN A form duplexes with their complementary segments, while non-complementary segments form rings, hairpins, and loops, which contain several nucleotides. The distances in these secondary structures differ from those between nucleotides in ordinary helices.

**Fig. 13:**
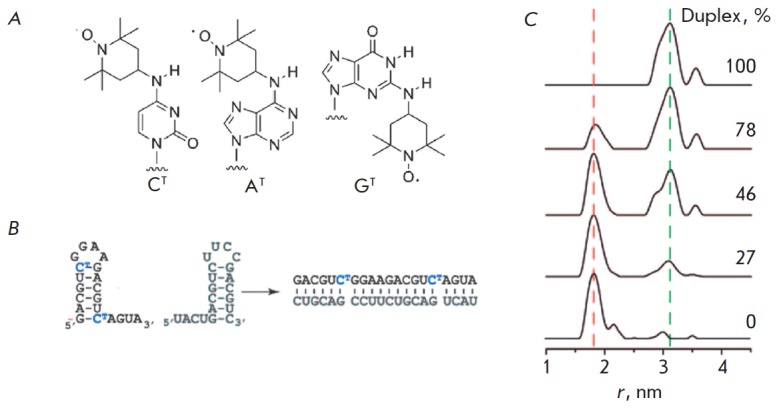
RNA hairpin structures investigated by PELDOR [67]. (A) RNA with spin-labeled nucleosides. (B) RNA duplex formation from two hairpins. (C) The distance spectrum in mixtures with different contents of the RNA duplex. (Reproduced by permission from John Wiley & Sons, Inc.: [Sicoli, G., Wachowius, F., Bennati, M., Hobartner, C. (2010) *Angew. Chem. Int. Ed. *49, 6443-6447], copyright 2010)

Data on the distances in the hairpin structure of spinlabeled RN A containing 20 nucleotides was obtained in [67]. Nitroxide TE MPO labels were introduced into the NH_2_ groups of guanine, adenine, and cytosine of certain nucleotides in single-stranded RN A (*Fig. 13A*). The number of nucleotides between the labels was fixed and equal to 10. The interlabel distance was determined using PELDOR during the formation of the complementary helix of spin-labeled and non-labeled RN A, as well as in the hairpin structures. In the first case, regardless of the nucleotide type and the position of the label pair in the RN A duplex, the interlabel distance remained the same and was equal to 3.1 nm, which corresponds to the calculated values for the A-form of the duplex.

In the RN A hairpin structure consisting of 20 nucleotides, six complementary nucleotides form a double helix (the hairpin stem), four nucleotides form the loop, and the remaining nucleotides form a monohelix. The duplex with labels located only in one strand was formed after a completely complementary RN A molecule without spin labels had been added to the system (*Fig. 13B*). The experiments with samples containing different amounts of spin-labeled RN A were carried out using PELDOR. The distance spectrum obtained in these experiments (*Fig. 13C*) attest to the existence of spin-labeled hairpins with a distance of 1.8 nm between the labels, as well as duplexes with a distance of 3.1 nm in frozen buffer solutions.

Single RN As can form hairpins along with more complex structures, such as rings (or semi-rings) in conjunction with hairpins. These structural elements may accept certain molecules; they are known as RN A riboswitches and play a crucial role in the mechanisms regulating the transfer of genetic information in cells.

Artificially synthesized RN A riboswitches consisting of 27 nucleotides and capable of accepting neomycin have been investigated using PELDOR [68]:

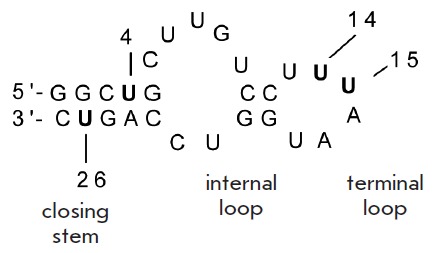



The TPA spin labels were introduced into uracil residues (highlighted in bold). The interspin distances between positions 4–14, 4–15, 14–26, 15–26 were determined. An attempt was made to determine the conformational changes in the RN A riboswitch during the formation of a neomycin complex [68]. It was found, however, that the distance spectrum between the spin-labeled uridine molecules virtually did not change in this complex as compared to the initial structure of the RN A riboswitch. According to [68], this fact suggests a conformational stability of the structure.

The structure of another enzyme capable of performing the functions of an RN A-riboswitch and/or of an RN A aptamer in respect to tetracycline (Tc) molecules was investigated [69]. Spin labels were introduced into the synthetic 57-mer ribooligonucleotide either via the reaction between (1-oxyl-2,2,5,5-tetramethylpyrroline- 3-methyl)methanethiosulfonate and 4-thiouridine or via the reaction between 4-isocyanate-2,2,6,6- tetramethylpiperidine-N-oxyl and the 2’-amino groups of the ribose molecule.

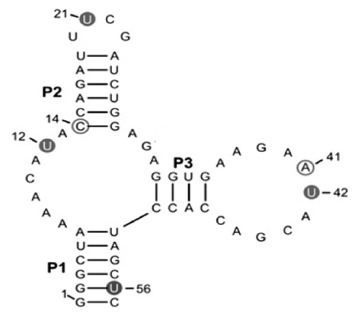



Here, the filled circles represent spin-labeled 4-thiouridine molecules and the hollow ones represent the nucleotides, which were spin-labeled at the 2’-amino groups.

The tentative structure of this RN A fragment contained three stems (P1, P2, and P3) and three loops. The distances between the spin labels incorporated at the positions (U12/U21), (U12/U56), (U42/U56), and (C14/A41) were measured using PELDOR in the absence and in the presence of Tc. It was determined that the free RN A aptamer exists in two different conformations. In the presence of the Tc ligand, an equilibrium shift towards one of the conformations occurs.

**Table 2:** Nucleic acids studied in [70]

Sample	Nucleotide sequence*
A hairpin (U6–U11)	5'-GGC-AC**U**-UC G-G**U**G-CC -3'
Neomycin riboswitch (U14–U26)	5'-GGC-UGC-UU G-UCC -U**U**U-AAU-GGU-CC A-G**U**C-3'
DNA duplex	5'-GCT -GAT-A**T**C-AGC-3'
	3'-CGA-C**T**A-TAG-TC G-5'

* Spin-labeled nucleotides are shown in bold.

The first attempt at using PELDOR to study the influence of the intracellular surrounding on spin-labeled DNA and RN A structures was reported in [70, 71]. In both works, which were published almost simultaneously, the behavior of spin-labeled nucleic acids was investigated in relatively large cells (~ 1 mm in diameter): oocytes of *Xenopus laevis*.

A 12-bp DNA duplex, a 14-mer RN A hairpin, and a 27-mer RN A riboswitch sensitive to neomycin were studied in [70] (*Table 2*). The TPA spin label was incorporated into the heterocyclic bases via the Sonogashira reaction. Approximately 50 cells were used for the PELDOR experiments. Each cell was infused with 30–50 nl of 2.5- to 5.0-mM free spin label or a double-labeled nucleic acid using microinjections. These manipulations took around 10 min. The cells were subsequently washed with a buffer, transferred into an EPR cuvette, and frozen in liquid nitrogen; then, the measurements were carried out.

The distances between the spin labels in the RN A hairpin and the neomycin RN A riboswitch did not depend on the localization of the specimen: whether inside the oocytes or outside the cell (in the buffer). This fact means that these RN A molecules had rigid structures, which are identical both *in vivo *and *in vitro*. In contrast, the interlabel distance in the short DNA duplex depended on the conditions experienced by the specimen: in the solution or inside the cell. The distance between the spin labels in the solution was smaller compared to that for a sample inside the cell. The authors attributed this fact to the existence of base stacking interactions when the duplex was localized in the solution and to stacking disruption when DNA was located inside the cell and interacted with cellular proteins and other molecules.

In [71], the TE MPA label was attached to the terminal residues of thymidine of each strand (shown in bold) in the DNA duplex via the Sonogashira reaction:

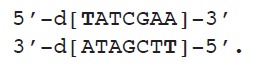



This spin-labeled duplex was introduced through a microinjection into the oocytes of *X. laevis*. The properties of spin-labeled DNA in cells frozen at 45 K and the physiological buffer solution were compared. In both cases, the concentration of paramagnetic particles after freezing decreased considerably: this did not hinder the assessment of the interlabel distance in DNA: 3.20 and 3.22 nm in buffer and inside the cells, respectively. The main impact consisted in a considerable increase in the width of the spectrum line. This value was equal to 0.43 nm in buffer and increased to 1.04 nm inside the cells. Based upon the presented PELDOR data, the latter value can be even higher; i.e. it can correspond to the virtually uniform spin distribution. This effect is presumably conditioned upon a relatively rapid degradation of DNA in the cellular environment, which occurs prior to freezing of the samples.

The increase in the stability of the spin labels in the cellular environment remains one of the main problems of this important research. Furthermore, it is possible also to reduce the time from the moment when a spinlabeled DNA is introduced into the cell to its freezing.

Numerous functional DNA and RN A molecules form specific tertiary structures, whose organization and dynamics determine their functions. The distances between the incorporated spin labels in these tertiary structures depend both on the biopolymer conformation and on the spatial orientation of its individual units.

The effect of Mg^2+^ ions on a ribozyme with a branched “hammerhead” structure (“hammerhead ribozyme,” HHRz) was also studied [72]. The distance spectrum for the TE MPO spin labels incorporated into various loops of the HHRz structure was determined. It was demonstrated that at the addition of Mn^+2^ ions into the HHRz solution, the number of ribozymes containing loops with the smallest interlabel distances (~ 2.4 nm) increases with the increase in Mn^+2^ concentration. It was assumed that these RN A–metal ion complexes participate in the catalyzed RN A cleavage.

The changes in the distances between the spin labels that are due to the conformational transformations were also estimated using PELDOR in other, more complex RN A and DNA molecules with various tertiary structures (e.g., RN A containing a threeway- junction) [73]. A similar structure is formed in the packaging motor of φ29-bacteriophage during the packaging of double-stranded genomic DNA into a preformed capsid. The packaging motor is an RN A-protein complex, which utilizes the energy from the ATP hydrolysis to condense genomic DNA. The structure of the RN A within this motor has yet to be studied thoroughly; hence, the significance of the investigation of its structure using PELDOR is not in doubt. This RN A is a dimer whose structure used to be regarded as a possible intermediate formed during this process. The R5 spin labels were introduced into the internucleotide phosphorothioate groups. A unit of this structure is shown below; the positions where the spin labels were incorporated are marked with asterisks:

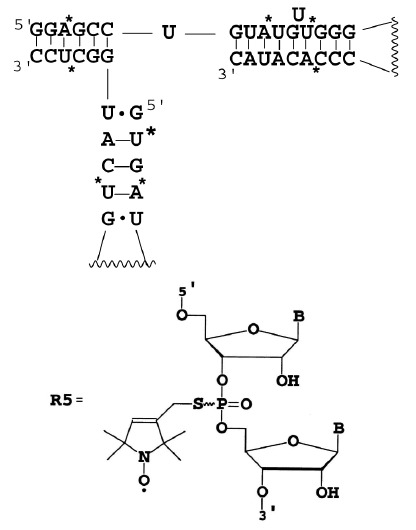



Seventeen distances between the spin-label pairs were measured. The analysis of these data allows one to determine the possible spatial orientations of three helices in the packaging motor. It was demonstrated that two out of three RN A helices in this structure formed a sharp angle with respect to each other, which does not correspond to the previously proposed model, where these helices were attached to each other along a single line. This work demonstrated all the advantages of the method used to study the spatial geometry of complex RN A structures.

**Fig. 14:**
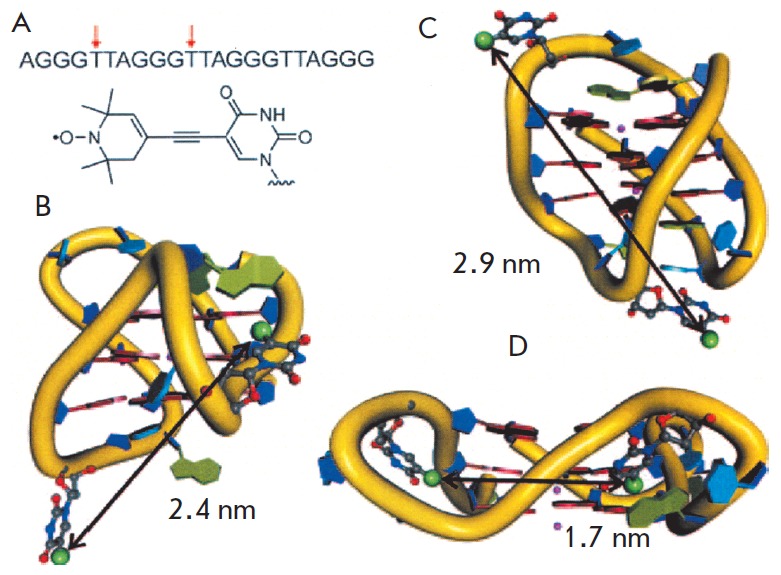
Human telomeric quadruplexes [74]. The DNA sequence was examined, arrows indicate the sites of the spin-labels in the conformers shown. Parallel propeller (A), hybrid 3 + 1 (B) and antiparallel basket (C). (Reproduced by permission from John Wiley and Sons: [Singh, V., Azarkh, M., Exner, T.E., Hartig, J.S., Drescher, M. (2009)* Angew. Chem. Int. Ed.* 48, 9728-9730], copyright 2009)

PELDOR was used to study the conformations of the quadruplexes formed in telomeric sequences at chromosome termini [74] (in humans, they consist of GGGTT A repeats [75]). The structure of the quadruplex- forming oligonucleotide double labeled with TE MPA, in which the spin labels were incorporated into positions 5 and 11, was studied (*Fig. 14A*) [74]. It has been proven that a mixture of two structures exists in a solution containing K^+^ ions: an antiparallel basket and a parallel propeller at a 1:1 ratio (*Fig. 14B,D*). Moreover, the sequence TT (GGGTT A)_3_GGGA, which is slightly different from the previous sequence, folded into a new hybrid 3+1 structure in the solution in the presence of K^+^ ions (*Fig. 14C*).

The four-way DNA junction (also known as the “Holliday junction”) and the changes in its structure during its interaction with endonuclease I of bacteriophage T7 were also investigated [76]. The DNA was made up of two strands: the Y strand (marked in red) and the Z strand (marked in blue), which formed a structure consisting of four different helices linked together at a single location:

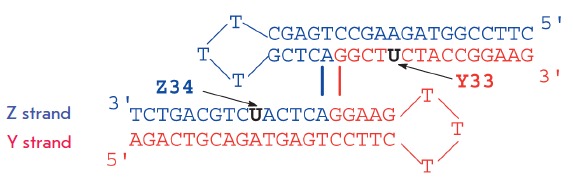



**Fig. 15:**
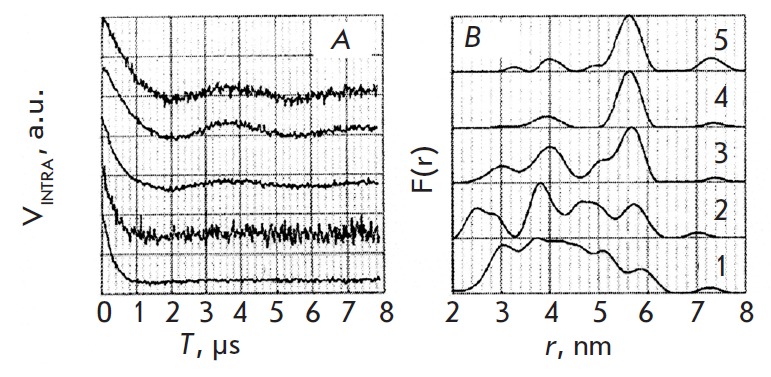
PELDOR time trace (A) and distance spectrum (B) changes as the function of the duplex/enzyme ratios in frozen buffer solutions: 1/0.00 (1), 1/0.25 (2), 1/0.50 (3), 1/1.00 (4), 1/1.25 (5) [76]. (Reproduced by permission from the American Chemical Society: [Freeman, A.D.J., Ward, R., Mkami, H.E., Lilley, D.M.J., Norman, D.G. (2011) *Biochemistry *50, 9963-9972], copyright 2011)

The TE MPO labels were introduced into the uridine residues of different duplex branches (the labels are denoted with the letter U and are highlighted in bold). The frozen buffer solutions contained D_2_O and deutero-glycerol. The PELDOR time traces were recorded and analyzed using the conventional procedures [21]. The result of endonuclease action on the four-way DNA junction is shown in *Fig. 15*. Prior to the introduction of the enzyme, a broad distance distribution between the TE MPO labels in the 3- to 6-nm range was observed. With enzyme concentration increasing (presumably due to the stabilization of the DNA–enzyme complex), the distance spectrum, with the interlabel distance in this complex being 5.6 nm, contained a single line (*Fig. 15*).

Data on the changes in the distances between the labels introduced into T7-endonuclease I during the formation of the duplex–enzyme complex were also obtained [76]. These changes in the distances, which occurred during the reorganization of the protein structure, were shown to be insignificant (not more than several A) but to be reliably recordable using PELDOR.

Thus, it has been reliably established that the induced fitting of the conformations in both biopolymers between T7-endonuclease I and the 4-way DNA-junction occurs during the complex formation. The conformational changes observed during the duplex– enzyme complex formation were confirmed using MD-simulations. It is worth mentioning that this work was characterized by high experimental quality (reliability and accuracy in the PELDOR experiment and its processing).

**Fig. 16:**
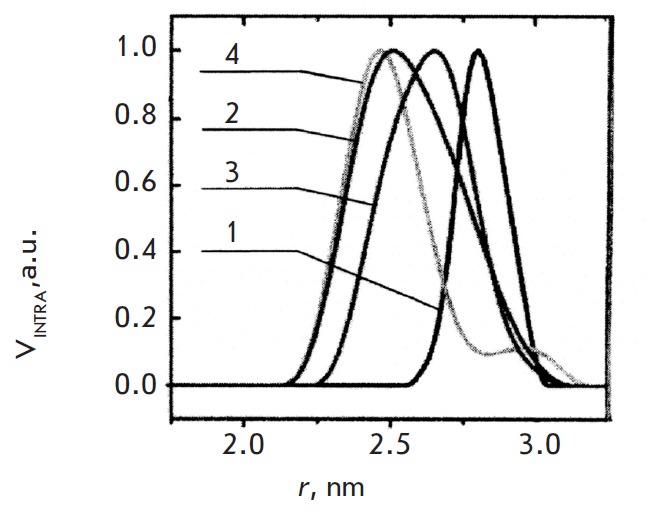
Distance distribution functions F(r) for undamaged DNA (1) and its changes when damages as propyl (2), ethyl (3), THF (4) (see Table 3) are introduced into DNA. Orientation selectivity was considered for F(r) calculated in the standard experiment [77]. (Reproduced by permission from Oxford University Press:[Sicoli,G., Mathis, G., Aci-Seche, S., Saint-Pierre, C., Boulard, Y.,Gasparutto, D., Gambarelli,S. (2009) *Nucleic Acids Res*. 37, 3165- 3176], copyright 2009)

**Effect of the deficiencies on the DNA structure**

The effect of various lesions modifying the DNA structure (in particular, changing the distances between the introduced spin labels) was studied in [77–79]. These defects can be caused by many factors, such as structural aggregates or groups binding DNA at a certain location, nucleotide substitution with various structures, nicks in one of the DNA strands in the duplex, etc. All these factors that chemically modify DNA become evident in the endogenous metabolism; their investigation employing physical methods is of particular interest for biologists and biochemists.

The changes in the interlabel distances in the DNA duplexes containing various structural defects and insertions in one of the duplex strands were analyzed in [77]. 4-Amino-TE MPO spin labels were introduced into the guanine residues of the other DNA strand (see reference [49]) into positions (4;11) (A) and (4;19) (B); the second strand of the duplex contained various lesions – nicks (C), gaps (D), modified nucleotides (E1–E5), and bulges (F) (*Table 3*).

4pPELDOR and conventional methods for data analysis were used in each case. The measurements were carried out at 60 or 70 K. The samples contained 50 or 100 μM of spin-labeled DNA in saline with 15–20% of glycerol added.

**Table 3:** Spin-labeled DNA duplexes with nicks and insertions [77]

A	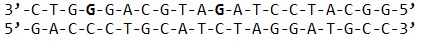	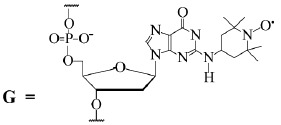
B	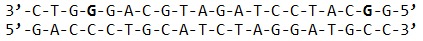	
C	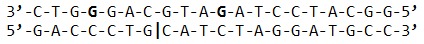	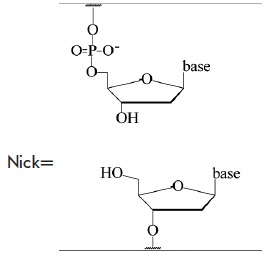
D	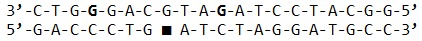	Gap
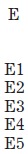	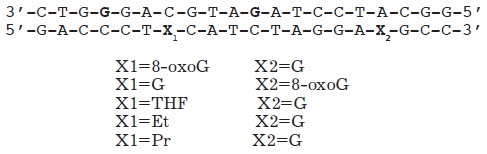	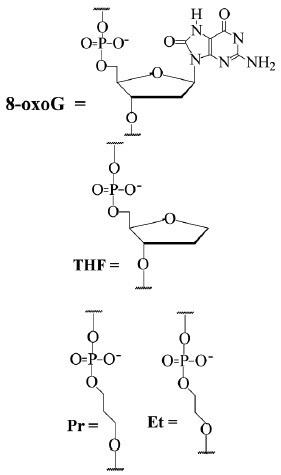
F	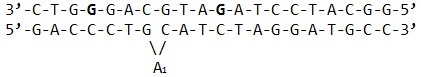	Insertion A_1_

The data for the undamaged duplexes (4;11) and for those with various lesions are provided in *Fig. 16* as an example of the distance spectra obtained from the modulated PELDOR time traces. In order to eliminate the orientational selectivity, the PELDOR time trace (averaged over 10 measurements) obtained via the variation of the magnetic field for the position of the detection pulse in the EPR spectrum was analyzed. The authors [77] believe that all these measures allow one to reliably assess the error in determining the average distance and the width of the lines in the distance spectrum (error ~10%) (*Table 4*).

**Table 4:** Experimental distances *r*_max_ between the spin labels and widths of the Δ-bands of the spectra lines (nm) in the duplexes with spin labels at positions (4;11) [77]

DNA duplex	*r*_max_	Δ
Undamaged duplex	2.81 ± 0.01	0.21 ± 0.02
Nick	2.87 ± 0.01	0.22 ± 0.02
Gap	2.84 ± 0.01	0.26 ± 0.02
Insertion А_1_	2.85 ± 0.02	0.23 ± 0.02
8-oxoG (E_1_ duplex)	2.81 ± 0.01	0.22 ± 0.02
8-oxoG (E_2_ duplex)	2.84 ± 0.01	0.27 ± 0.02
THF	2.46 ± 0.02	0.35 ± 0.02
Ethyl	2.65 ± 0.02	0.38 ± 0.04
Propyl	2.48 ± 0.02	0.45 ± 0.03

All the results obtained for the duplexes labeled at positions (4;11) were separated into two groups [77]. The first group contained the duplexes with structural lesions of C, D, F, E1 and E2 types. The changes in the interspin distances in this group were insignificant in comparison with the undamaged DNA and were mostly due to measurement errors. A significant decrease in the distance, along with widening of the distance spectrum lines leading to its asymmetry, was found in the second group, which contained duplexes with E3, E4, E5 lesion types. A similar situation was observed for the duplexes labeled at positions (4;19), where the distances change from 5.21 ± 0.04 nm for the initial, undamaged structure to 5.02 ± 0.03 nm for the damaged duplexes of (5.4), (5.5) type; the width of the distribution function changes from 0.33 ± 0.02 to 0.44 ± 0.05 nm.

When discussing the results, it is usually assumed that the width of the distance spectrum line characterizes the conformational flexibility of the duplexes. Following special MD calculations, G. Sicoli *et al. *[77] concluded that the significant changes in the distances in the second group of damaged duplexes could be attributed to the local changes in the conformations at lesions sites and in the complementary nucleotide of the duplex.

In general, the results in study [77] (where the changes in the damaged DNA were examined) in a number of cases are in qualitative agreement with the data obtained using such methods as NMR. It is also considered that the use of pulse EPR spectroscopy in combination with MD techniques for spin-labeled DNA is complementary (to conventional methods, such as NMR, CD, FRET , and X-ray crystallography) and a highly informative method for studying various DNA lesions and weak interactions between DNA and other molecules and complexes.

The sensitivity of the PELDOR parameters determined to the changes in the nucleic acid structure was found to be considerably higher than that in [77], when the spin labels were introduced into the termini of relatively short nucleotides and their duplexes [78, 79].

3pPELDOR was used to identify the distance spectrum for the labels at the termini of 12-mer oligonucleotides and their DNA duplexes [78]. The synthesized substrates contained TE MPO spin labels at their 5’- and 3’- terminal phosphate groups.

The structures of 12-mer single-stranded DNA and DNA duplexes, as well as their denotation, are provided below:

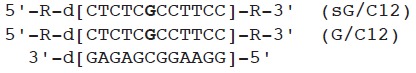



One of the nucleotides located in the center of the G strand could be modified by various insertions and substitutions. The structures of the spin labels and introduced insertions are listed in *Table 5*.

**Table 5:** Structures of radical R, nucleotides, and non-nucleotide insertions [78, 79]

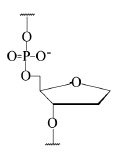	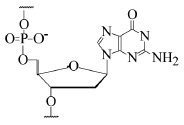	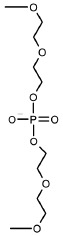	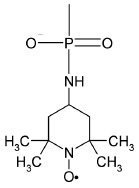	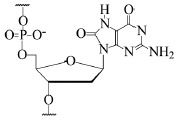
THF	G	deg_2_p	R	8-oxoG

**Fig. 17:**
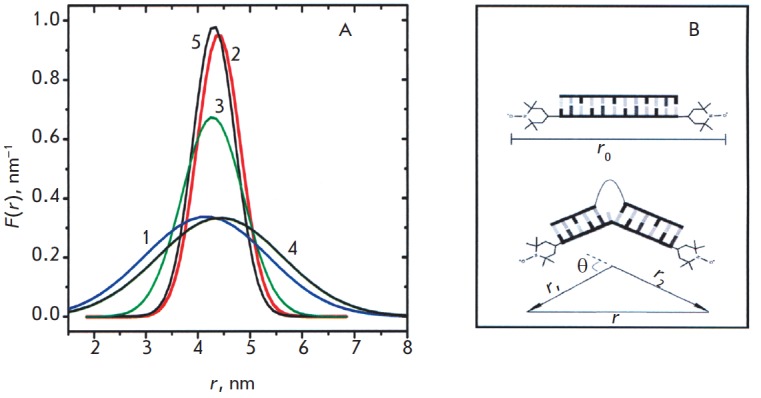
(A) Gaussian approximations of the spectra lines for two spin labels in DNAs in a frozen glassy water/glycerol mixture at 77 K [78]. (1) – single-stranded oligonucleotide ssG, (2), thick line – duplex dsG, (3 – duplex dsG-looped, 4 – single-stranded oligonucleotide ssF, 5 –duplex dsF. (B) Schematic representation of the spin-labeled DNA molecule without (top) and with (bottom) a non-nucleotide insert, which demonstrates the bending of the molecule and shortening of the distance between two labels. **(**Reproduced by permission from The Royal Society of Chemistry: [Kuznetsov, N.A., Milov, A.D., Koval, V.V., Samoilova, R.I., Grishin, Yu.A., Knorre, D.G., Tsvetkov, Yu.D., Fedorova, O.S., Dzuba, S.A. (2009) *Phys. Chem. Chem. Phys. *11, 6826-6832], copyright 2009)

Frozen glassy solutions of spin-labeled DNA were studied in a water/glycerol mixture at 77 K using 3pPELDOR in order to obtain the distance spectrum between the labels. These distance spectra were determined from the experimental *V_INTRA_*time traces by the Tikhonov’s regularization method with the use of both the standard algorithm and the Gaussian approximation for *F(r*).

The distance spectra shown in *Fig. 17A* and the data listed in *Table 6* demonstrate that a 2- to 3-fold narrowing of the spectrum lines as compared to those for single-stranded DNA occurs during the duplex formation. The insertion of nucleotide analogues results in a reduction in the average interspin distance in the duplexes. In case of the **deg_2_p **insertion, a noticeable widening of the distance spectrum as compared to **G/ C12 **duplexes was observed. It is obvious that the line narrowing of the distance spectrum can be attributed to the formation of the DNA double helix, which is characterized by a more rigid structure as compared to that of single-stranded DNA. The observed width of the spectrum line for the undistorted duplex (**G/ C12**) was apparently caused by the random orientation of spin labels due to rotation around the P–N bonds. Considering the fact that the distance between the nitrogen atom and the N–O moiety of the spin label is ~ 0.4 nm, the maximum widening of the spectrum line due to the reorientation of spin labels will be equal to 1.6 nm. The magnitude of the experimental value of the width of the distance spectrum Δ = 0.98 ± 0.1 nm for the undamaged duplex lies within this range. It should be mentioned that the effects associated with the orientational selectivity in PELDOR were observed neither in this study nor in [77], which can also presumably be attributed to the uncorrelated spread of spin label orientations.

**Table 6:** Parameters of the distance spectra (nm) for 12-meric oligonucleotides and their DNA duplexes ;[78]

Sample^*^	Average distance, r	Width, Δ
sG/C12	4.05 ± 0.05	2.8 ± 0.2
sTHF/C12	4.32 ± 0.05	2.85 ± 0.2
G/C12	4.35 ± 0.03	0.98 ± 0.1
deg_2_p/C12	4.23 ± 0.03	1.39 ± 0.1
THF/C12	4.26 ± 0.03	0.95 ± 0.1

* Symbol **s **denotes the single-stranded DNA.

The introduction of non-nucleotide insertions into the duplex structure affects the average interspin distance. A decrease in the distance denotes the possibility of duplex bending with respect to the insertion sites, due to the emergence of an additional degree of freedom in the insertion site. The scheme illustrating the estimation of the conformational distortion angle is shown in *Fig. 17B*. The experimentally determined values of *r*_0_ (the duplex without the insertion) and *r *for the distorted duplex can be used to estimate the angle (θ = 23˚) in the **ТНF/C12 **duplex and (θ = 27˚) in the looped **deg_2_p/C12 **duplex.

An increase in the length and flexibility of the insertion in the **deg_2_p/C12 **duplex as compared with those in the other two duplexes results in additional line broadening (Δ = 1.39 nm) of the distance spectrum. The broadening value is too large to simply attribute it to the spread in the bending angle of the duplex without taking into account the possible elongation of the duplex.

Hence, the aforementioned estimation of the bending angle of this insertion can only be used as the lower boundary of the average bending angle for this duplex. Therefore, the provided estimates show that the incorporation of a non-nucleotide insertion into a DNA molecule opens the possibility of duplex bending with respect to the insertion site. An increase in the number of bonds in the insertion increases insertion flexibility. A decrease in duplex rigidity near the insertion causes a considerable increase in the dispersion of the bending angle and the total duplex length.

**Fig. 18:**
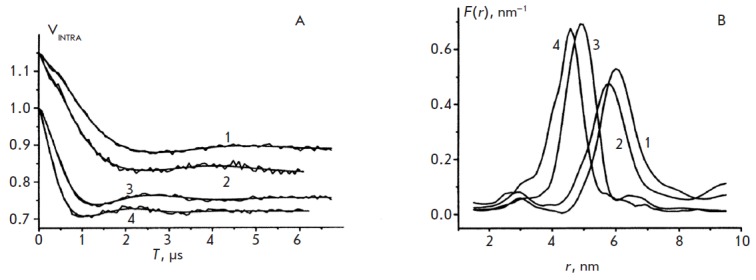
The intramolecular contribution to the PELDOR signal obtained for complexes of DNA with Fpg [79]. (A) Curves 1 and 2 refer to DNA G/C^17^ and F/C^17^/Fpg, respectively; curves 3 and 4 refer to DNA G/C^13^ and F/C^13^/Fpg, respectively. For convenience of comparison, curves 1 and 2 are shifted upwards relative to curves 3 and 4. The smooth curves were calculated using the distribution functions shown in Fig. 18B. (B) The distance distribution function between labels, *F(r), *obtained from the data in Fig. 18 (A) neglecting the orientational selectivity. Curves 1 and 2 refer to DNA G/C^17^ and F/C^17^/Fpg, respectively; curves 3 and 4 refer to DNA G/C^13^ and F/C^13^/Fpg, respectively.** (**Reproduced by permission from The Royal Society of Chemistry: [Kuznetsov N.A., Milov A.D., Isaev N.P., VorobjevYu.N., Koval V.V., Dzuba S.A., Fedorova O.S., TsvetkovYu.D. (2011) *Mol. BioSystems *7, 2670-2680], copyright 2009)

N.A. Kuznetsov *et al. *[79] used the same approach as that used in [78] to study the lesions in stretched DNA duplexes (13 and 17 nucleotides). However, the main aim of this work was to investigate the features of the conformational transformations of DNA during its interaction with the Fpg protein from *E.coli*. This protein is considered to be one of the key factors involved in the process of DNA repair. The structure of the investigated duplexes is shown below:

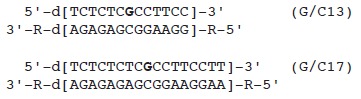



The structures of the R spin labels, nucleotides, and non-nucleotide insertions are presented in *Table 5*.

The analysis of the PELDOR time traces (*Fig. 18A*) allowed to obtain the distance spectra (*Fig. 18B*) and to determine the structural parameters: the distance values at the maximum of the distribution lines, *r*max (0.8% accuracy), and the width of the lines at half-height Δ (10% accuracy) (*Table 7*).

Similar to that in [78], the positions of the spin labels at the 5’- and 3’- terminal ends of the complementary second oligonucleotide of the DNA duplex made them sensitive to the formation of the DNA curves caused by either the existence of damaged sites or the formation of complexes with an enzyme. The Fpg protein from *E. coli *was found to cause bending even in the undamaged 13-bp duplex. A similar result has been obtained only with the use of an X-ray structure analysis of Fpg from* Bacillus stearothermophilus* [80–82]. However, no bend formation has been detected for the undamaged 17-bp duplex in the presence of the enzyme. This could be attributed to the fact that the enzyme occupying a 10-bp DNA segment cannot move (slide) along the strand of the short DNA duplex, while sliding is possible during binding to the 17- bp DNA duplex. It cannot be ruled out, however, that the conformational mobility of the spin labels of the 17-bp duplex is higher than that of the 13-bp duplex, which is supported by an increase in the width of the spectrum line for the 17-bp duplex (see *Table 7*).

In free DNA duplexes containing 8-oxoG, changes in the interspin distances as compared with the undamaged duplex have not been identified. This is not surprising, since 8-oxoG hardly changes the DNA structure. A considerable reduction in the interspin distance was observed for the duplexes containing the cyclic THF site. This result was confirmed by computer simulations using the molecular dynamics method [79].

During the interaction between the duplexes and the Fpg protein from *E. coli*, bending occurred both in 13- and 17-unit duplexes. This result correlates with the X-ray structural data for the cross-linked adduct of Fpg and the apurinic/apyrimidinic site [80–82]. It is important to mention that the X-ray data provide information on the local DNA segments in the damaged region, whereas PELDOR provides data on the global changes in the structure. Hence, structural studies of both types agree with and complement each other.

The bending of the DNA helix in the region of the damaged nucleotide recorded using PELDOR [78, 79] provides new information regarding the mechanism of the search for lesions in DNA by DNA repair enzymes. The emergence of the bendings allows one to understand why the enzymes that slide along the DNA strand stop at the damaged sites to repair them. The data obtained are also important for understanding the mechanisms of action of other enzymes that perform the search for specific DNA sites.

## CONCLUSIONS

Let us summarize the results of these PELDOR studies of DNA and RN A. It has been demonstrated that the PELDOR method can be used to determine DNA and RN A conformations and the conformational changes that are due to structural modifications and transformations, to assess the dispersion in the distances between individual groups and structure rigidity, and to determine the orientation of the spin labels introduced into DNA and RN A by measuring the distances between various fragments of poly- and oligonucleotides. Studies of the effect of the surrounding environment and complex formation on the structural parameters of DNA and RN A have been launched.

The studies summarized in this review describe the features, advantages, and drawbacks of PELDOR as compared with similar structural methods. Among the most obvious and most important advantages, let us mention in particular the relatively wide range of distances between the spin labels (1.5–8 nm) measured with high accuracy. The fact that it is not only the distances but also the distance distribution spectra that can be determined makes PELDOR prominent among the other structural methods. Relatively simple methods for analyzing the experimental data obtained for randomly oriented systems, in mixtures and solutions (which are convenient media for chemists and biologists), have been elaborated. Pulse EPR spectrometers are commercially available. All these factors determine the popularity of PELDOR in modern chemical radiospectroscopy, especially for biologically important systems.

**Table 7:** Parameters (nm) of the distance distribution function* F*(*r*) between two spin labels in the DNA duplexes [79]

Sample	*r*_max_, nm*	Δ, nm*
G/C13	4.96	1.1
8-oxoG/C13	4.96	1.1
THF/C13	4.83	1.1
THF/C13/Fpg	4.60	1.2
G/C13/Fpg	4.78	1.1
G/C17	6.00	1.2
8-oxoG/C17	6.02	1.2
THF/C17	5.98	1.2
THF/C17/Fpg	5.76	1.2
G/C17/Fpg	5.99	1.4

* *r*_max_ and Δ were measured with errors of 0.8 and 10%, respectively.

Meanwhile, we attribute the necessity of introducing spin labels into the investigated molecule to the disadvantages of this method as compared with such methods as NMR. However, despite the fact that a series of chemical methods have been developed, site-directed spin labeling still remains a relatively labor-consuming manipulation. Moreover, one needs to make sure that a spin-labeled molecule does not lose its initial physicalchemical properties. Because of the “molecule–label” effects of a linker, the measured interspin distance can differ from the molecular distance. All these problems have typically existed and were solved in one way or the other in most of the aforementioned works. One also needs to select a special solvent that has to undergo glassing during freezing, since all the measurements are carried out in glassy matrices at low temperatures. We would like to point out that the same disadvantages were encountered when measuring the distances using the dipole widening of the lines in a stationary EPR spectrum confined to distances of less than 2–2.5 nm [29].

In methodological terms, PELDOR is similar to the commonly used FRET method [83], which helps study the excitation transfer between the incorporated donor and acceptor labels. It is worth mentioning that the two fluorescent labels, which are introduced to carry out FRET measurements, differ in their structures (as opposed to similar labels for the PELDOR method) and are typically relatively larger as compared to spin labels. The efficiency in FRET is proportional to the 1/ [1 + (*r*/*R*_0_)^6^] value, where *R*_0_ is the Forster radius and* r *is the interlabel distance [84]. The Forster radius depends on a number of parameters, such as the overlap integral of the donor spectrum with the acceptor one, the fluorescence quantum yield, and the orientation of electrical dipoles. Unlike that in PELDOR, all these factors require additional experiments and calibrations in order to determine the interlabel distances. This reduces accuracy in determining these distances. The extremely high sensitivity of FRET is its major drawback. Similar to most modern optical methods, it can be used to perform measurements with a resolution of up to single molecules, including quick transformations in liquids. PELDOR has a maximum sensitivity of approximately 10^12^ particles per sample [10].

Hence, the choice of the investigation method to study the structure and properties of poly- and oligonucleotides is determined primarily by the aims of the study and experimental capabilities. Ideally, a combination of PELDOR and FRET provides the most comprehensive information on the structure and physical-chemical properties of biologically important structures. Such studies are currently under way. For instance, studies employing these methods to investigate the features of the protein–nucleic complexes structure have already been published [85, 86]. Among them, a relatively complex supramolecular complex regulating the structure of chromatin histones has been studied [87].

In our opinion, the data obtained with the use of PELDOR significantly contributes to the investigation of the structure and properties of DNA and RN A. This method opens new perspectives for studying complex nonlinear structures, interactions between polynucleotides and enzymes, proteins, and membranes. The potential of PELDOR as a method for structural studies will undoubtedly increase with the development of pulse ER P spectroscopy.
